# Impact of a structured urine culture request form on antimicrobial stewardship in urinary tract infections at a tertiary care hospital in India

**DOI:** 10.3389/frabi.2026.1793784

**Published:** 2026-06-01

**Authors:** Supreeta R Shettar, Mahadevaiah Neelambike Sumana, Manjunath S Shetty, Yogeesh D Maheshwarappa, Raghukanth Reddy, Asha Srinivasan, Gautam Kalyatanda, Amruthraj G Gowda

**Affiliations:** 1 Department of Microbiology, JSS Medical College and Hospital, JSS Academy of Higher Education and Research (AHER), Mysuru, India; 2 Deptartment of Nephrology, JSS Medical College and Hospital, JSS Academy of Higher Education and Research (AHER), Mysuru, India; 3 Division of Nanoscience and Technology, School of Life Sciences, JSS Academy of Higher Education and Research (AHER), Mysuru, India; 4 Division of Infectious Disease and Global Medicine, Department of Medicine, Gainesville, FL, United States; 5 Department of Urology, JSS Medical College and Hospital, JSS Academy of Higher Education and Research (AHER), Mysuru, India

**Keywords:** antimicrobial stewardship, asymptomatic bacteriuria, diagnostic stewardship, India, multidrug-resistant organisms, urinary tract infection, urine culture

## Abstract

**Background:**

Interpretation of urine culture results requires key clinical information such as symptom status, catheterization, and multidrug-resistant organism (MDRO) risk. However, this information is frequently absent from routine request forms, contributing to overtreatment of asymptomatic bacteriuria (ASB) and suboptimal antimicrobial prescribing. This study evaluated the impact of implementing a structured urine-culture request form as part of a diagnostic and therapeutic stewardship program.

**Methods:**

This prospective, non-randomized interventional study was conducted at an 1800-bed tertiary-care hospital in India (March 2023–March 2024). Patients undergoing urine culture testing in Urology/Nephrology departments used a structured request form capturing symptoms, risk factors, and clinical context (test arm), while General Medicine continued routine forms (control arm). Primary outcomes included ASB treatment, MDRO detection, recurrence, and guideline-concordant prescribing. Patients were followed for one year.

**Results:**

A total of 484 patients were included (198 test arm, 286 control arm). Antibiotic treatment for ASB was significantly lower in the test arm compared with the control arm (3.6% vs 67.0%; p < 0.001), without adverse outcomes. Guideline-compliant prescribing was higher in the test arm (73.7% vs 26.2%; p < 0.001). MDRO prevalence was higher in the test arm (32.2% vs 11.3%), reflecting greater clinical complexity rather than the intervention itself. Recurrent urinary tract infection (UTI) within one year was significantly lower in the test arm (14.1% vs 29.0%; p < 0.001).

**Conclusions:**

Introducing a structured urine-culture request form improved diagnostic clarity and antibiotic prescribing, particularly by reducing unnecessary treatment of ASB and increasing guideline compliance, without compromising patient outcomes. This low-cost intervention represents a practical and scalable diagnostic stewardship strategy for improving UTI management.

## 1 Introduction

Urinary tract infections (UTIs) are among the most common bacterial infections worldwide and a leading cause of empirical antimicrobial prescriptions (Flores-Mireles et al., 2015). Although urine culture remains the diagnostic gold standard, its clinical interpretation requires essential contextual information such as symptoms, catheter status, and risk factors for multidrug-resistant organisms (MDROs) (Wilson and Gaido, 2004; Sinawe and Casadesus, 2025). Conventional urine culture request forms often lack these details, creating challenges for clinical microbiologists in distinguishing true infection from colonization or contamination (Flokas et al., 2017).

The absence of relevant clinical details often results in inappropriate reporting of insignificant bacteriuria, mismanagement of asymptomatic bacteriuria (ASB), and failure to prioritize higher-risk patients for appropriate sensitivity testing (Leis et al., 2014; Flokas et al., 2017). ASB, which can constitute up to 60% of positive urine cultures in some hospital settings, is frequently over treated due to misinterpretation of laboratory results in the absence of clinical context (Nicolle, 2006). Treatment of ASB in patients without specific risk factors such as pregnancy or urological interventions offers no clinical benefit but contributes to unnecessary antibiotic use and fosters antimicrobial resistance (Nicolle et al., 2005; Nicolle et al., 2019). Studies report overtreatment rates of ASB exceeding 70% in hospitalized patients, underscoring the need for interventions that prevent misdiagnosis at the laboratory interface (Petty et al., 2019).

Failing to identify patients with MDRO risk factors at the time of urine culture request delays targeted susceptibility testing, leading to under detection of resistant pathogens and suboptimal empirical treatment (Tamma et al., 2014). Accurate identification of MDROs is critical, given the resistance rates among common uropathogens such as *Escherichia coli* and *Klebsiella pneumoniae* continue to rise globally (Logan and Weinstein, 2017).

Antimicrobial stewardship programs (AMSPs) promote optimizing antibiotic use; however, most interventions primarily target prescribers rather than laboratory processes (Barlam et al., 2016). Diagnostic stewardship, which focuses on optimizing test ordering, processing, and reporting, remains underutilized in routine clinical microbiology (Morgan et al., 2017). Previous diagnostic stewardship efforts have largely centered on restricting unnecessary urine cultures or introducing reflex urine microscopy screening (Lee et al., 2021). Yet, few interventions addressed the critical pre-analytical gap: the lack of clinical information accompanying urine culture requests.

Structured urine culture request forms that capture essential clinical data such as patient symptoms, catheterization status, pregnancy, recent hospitalization, or immunosuppression have the potential to guide clinical microbiologists and laboratory professionals in more accurate interpretation of culture results, selectively reporting of susceptibility patterns, and reducing unnecessary treatment of ASB (Rawson and Moore, 2023). Despite this, their adoption in routine hospital workflows remains limited, and data evaluating their impact are scarce (Rawson and Moore, 2023).

In this study, we introduced a structured urine culture request form to address this gap in diagnostic and therapeutic stewardship. The form, designed collaboratively with clinicians and microbiologists, incorporated key clinical parameters to support targeted culture processing, appropriate antibiotic susceptibility reporting, and early MDRO detection. This study aimed to assess the impact of this intervention on microbiological reporting practices, antibiotic prescribing, ASB management, MDRO identification, and patient outcomes.

## 2 Methodology

### 2.1 Study design and setting

This prospective, non-randomized interventional study was conducted at JSS Medical College and Hospital, Mysuru, India, an 1800-bed tertiary care teaching hospital from March 2023 to March 2024. Ethical approval was obtained from the Institutional Ethical Committee (Approval No: JSSAHER/REG/RES/URG/54/2023-24/14906). Written informed consent was obtained from all participants or their legal representatives for participation and one-year follow-up.

### 2.2 Sample size

A formal sample size calculation was not performed, as this was a pragmatic, implementation-based interventional study designed to include all eligible patients over a fixed one-year period. However, the final sample size was comparable to or larger than similar stewardship studies, and *post hoc* evaluation demonstrated adequate statistical power for the primary outcomes.

### 2.3 Study population

Patients of any age and gender presenting with suspected UTI and undergoing urine culture and sensitivity testing from either the Urology/Nephrology departments (test arm) or the General Medicine department (control arm) were included in the study. Patients who did not provide informed consent or whose urine samples were improperly collected or deemed unprocessable were excluded.

### 2.4 Study groups and intervention

In the test arm (Urology and Nephrology departments), a newly designed structured urine culture request form was implemented as part of a diagnostic and therapeutic stewardship program. The form was developed collaboratively by clinical microbiologists, infectious disease specialists, and nephrologists to address key challenges in urine culture interpretation and antimicrobial stewardship. It incorporated essential clinical parameters including symptom status to differentiate symptomatic urinary tract infection from ASB, risk factors for MDROs such as recent hospitalization, prior antibiotic exposure, post-renal transplant status, and duration of catheterization, as well as special clinical conditions influencing ASB management, including pregnancy status and recent or planned urological procedures. In addition, the form captured the source of urine sample (midstream, catheterized, suprapubic, or straight catheter) with corresponding thresholds for significant bacteriuria, and included details of recent antibiotic or diuretic use to guide interpretation of low-count bacterial growth. A detailed summary of these parameters and associated microbiological reporting strategies is provided in **Supplementary Table 1**, and the complete request form is included as **Supplementary Figure 1**.

The structured form enabled clinical microbiologists to tailor laboratory processing and reporting according to clinical context. This included modification of processing protocols for low-count organisms, selective reporting of antimicrobial susceptibility results based on infection type (for example, restricting nitrofurantoin reporting in upper urinary tract infections), and provision of treatment guidance aligned with current Infectious Diseases Society of America (IDSA) and Indian Council of Medical Research (ICMR) 2022 guidelines. Prior to implementation, structured educational sessions and workshops were conducted for clinicians in the test arm to ensure appropriate completion of the form and understanding of its stewardship objectives. Educational sessions and periodic reinforcement were conducted to promote adherence to form completion, which ensured high uptake in the test arm, as reflected by completeness of clinical documentation.

**Figure 1:**
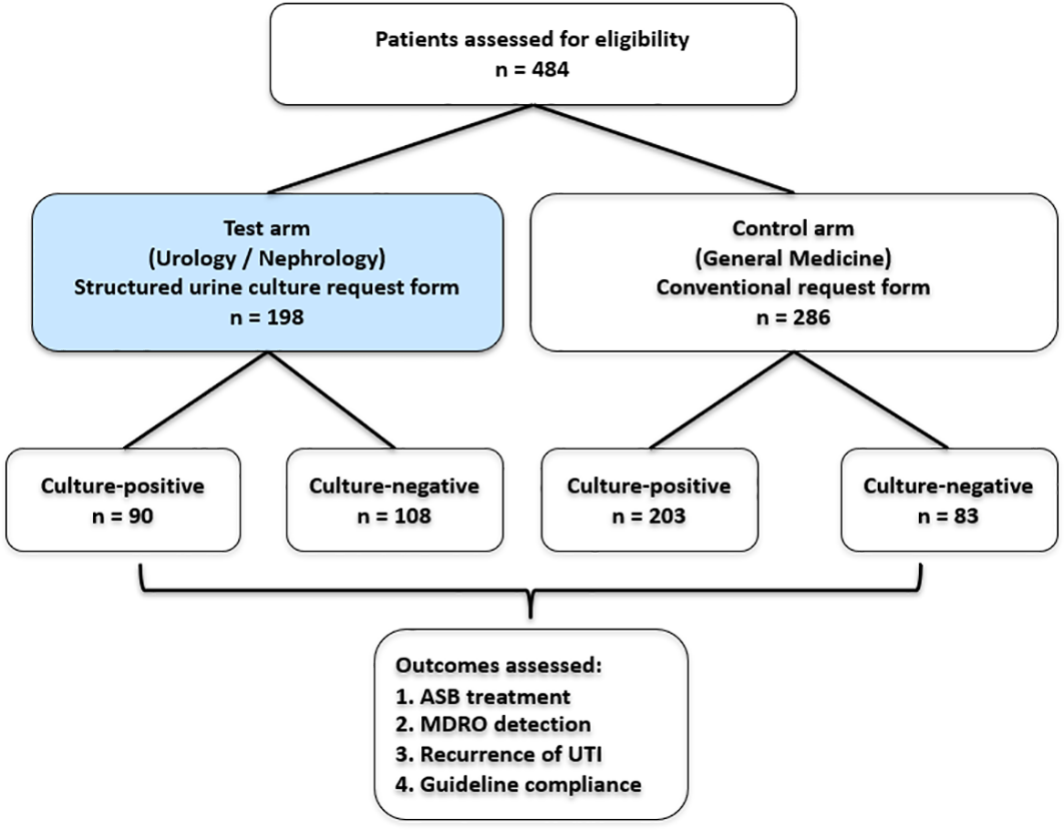
Study design and patient flow.

In the control arm (General Medicine department), conventional urine culture request forms were used. These forms included only basic demographic information, clinical diagnosis, and details of ongoing antibiotic therapy. Urine cultures in this group were processed and reported according to standard laboratory protocols without the application of diagnostic or therapeutic stewardship interventions. **Figure 1** shows Study design and patient flow of the study.

### 2.5 Sample collection and laboratory methods

Urine samples were processed according to standard microbiological protocols. Wet mount microscopy was performed to assess pyuria, with >5 neutrophils per high-power field considered significant. All samples were cultured on HiCrome UTI HiVeg agar.

Antimicrobial susceptibility testing was performed using the VITEK-2 Compact system, employing GP-AST628 cards for Gram-positive organisms and AST N405/N406 cards for Gram-negative organisms. The AST N407 card was additionally used in cases of suspected multidrug-resistant infection or sepsis. Nitrofurantoin susceptibility testing was performed manually in accordance with Clinical and Laboratory Standards Institute (CLSI) M100 guidelines.

### 2.6 Outcome measures

The primary outcomes included reduction in unnecessary antibiotic use for ASB, early detection of MDROs, recurrence of UTI within one year, and compliance with recommended antimicrobial treatment guidelines. Secondary outcomes included duration and dosage of antibiotic therapy, as well as antibiotic consumption cost, which was specifically analyzed in patients with uncomplicated UTIs to ensure comparability between study arms.

### 2.7 Compliance monitoring and follow-up

Compliance defined as adherence to IDSA/ICMR treatment guidelines concerning choice, dose, duration, and route of antimicrobials.

Patients were followed for one year using outpatient records and telephonic interviews to assess treatment adherence, recurrence, and clinical outcomes. A copy of the follow up form is included in the **Supplementary Figure 2**.

### 2.8 Statistical analysis

Statistical analysis was performed using standard analytical procedures used in SPSS/R. Categorical variables were summarized as frequencies and percentages and compared using the χ² test (and Fisher’s exact test only when an expected cell count was <5). Continuous variables were assessed for normality using the Shapiro–Wilk test. Normally distributed variables were compared using the independent-samples t-test, and non-normally distributed variables were compared using the Mann–Whitney U test.

Because baseline characteristics differed between study arms, multivariable regression models were applied. Logistic regression was used for binary outcomes (culture positivity, ASB overtreatment, MDRO isolation, recurrence and guideline compliance), and linear regression for continuous outcomes (duration of therapy and antibiotic cost). All models were adjusted for age, sex, catheterization, recent hospitalization and comorbidity/complicated UTI status. Effect sizes are reported as adjusted odds ratios (AOR) or β-coefficients with 95% confidence intervals (95% CI). A two-sided p < 0.05 was considered statistically significant.

## 3 Results

### 2.1 Demographics and baseline characteristics

A total of 484 patients undergoing urine culture testing were included, of whom 198 (40.9%) were managed in the test arm (urology/nephrology departments using the structured urine-culture request form) and 286 (59.1%) in the control arm (general medicine using the routine form). Mean patient age differed slightly between study arms, being 50.97 ± 16.34 years in the test arm and 48.72 ± 16.68 years in the control arm. Age was not normally distributed in either group (Shapiro–Wilk p < 0.001 for both); therefore, between-group comparison was performed using the Mann–Whitney U test, which showed no statistically significant difference (p = 0.16).

Gender distribution differed significantly. Females comprised 183/286 (64.0%) of the control arm compared with 90/198 (45.5%) of the test arm (χ² p < 0.001). Culture positivity was also significantly higher in the control arm (203/286; 71.0%) than in the test arm (90/198; 45.5%) (Fisher’s exact p < 0.001) as provided in **Table 1**. These baseline differences were accounted for in later adjusted regression models.

**Table 1:** Baseline demographic characteristics and culture positivity.

Characteristic	Test arm (n=198)	Control arm (n=286)	p-value
Mean age ± SD (years)	50.97 ± 16.34	48.72 ± 16.68	0.16¹
Female sex	90 (45.5%)	183 (64.0%)	<0.001²
Culture-positive samples	90 (45.5%)	203 (71.0%)	<0.001³

¹Mann–Whitney U test, ²Chi-square test, ³Fisher’s exact test.

### 2.2 Pathogen distribution and MDROs

**Figure 2:**
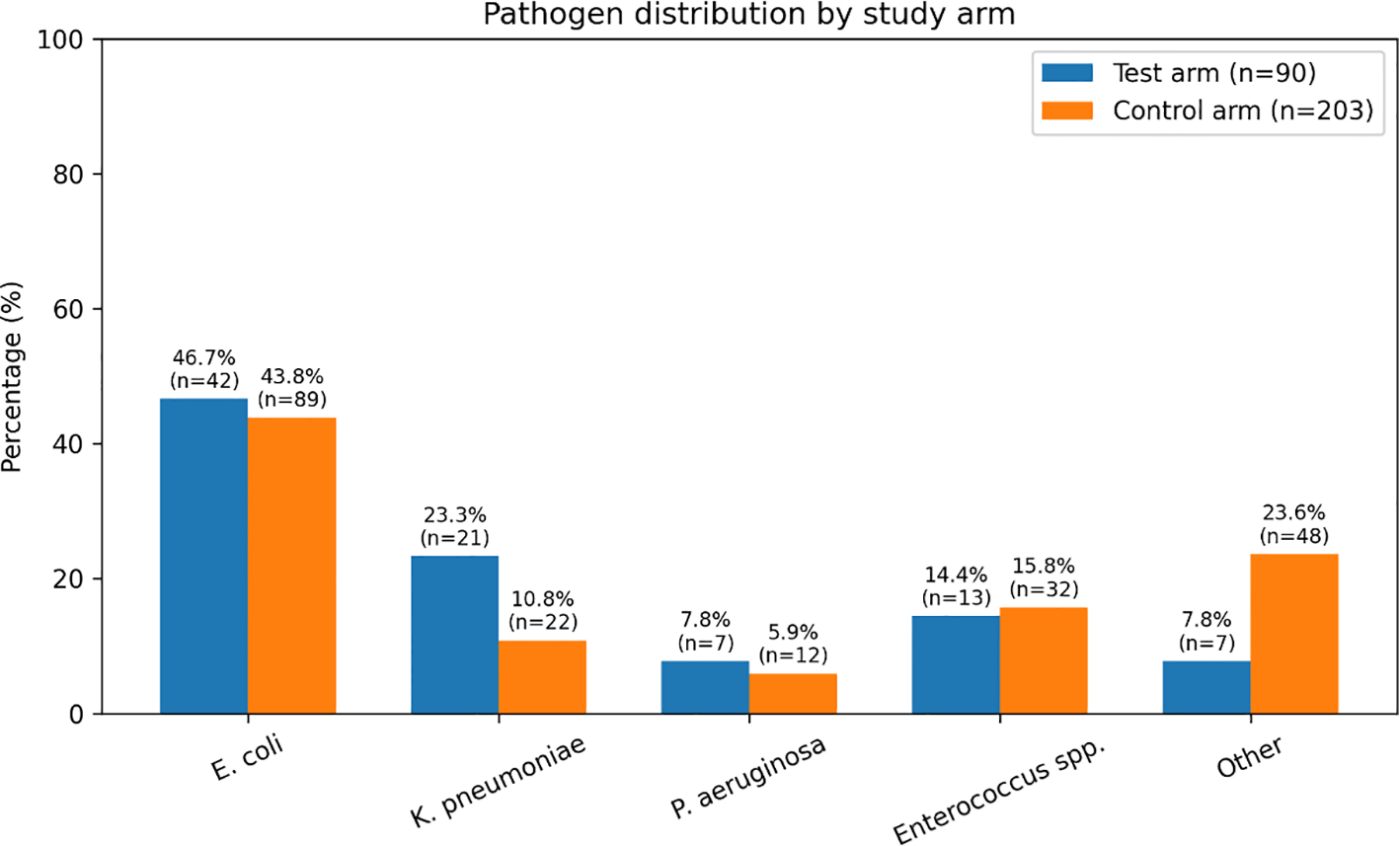
Pathogen distribution by study arm.

Among the 293 culture-positive urine samples, *Escherichia coli* was the most frequently isolated pathogen in both study arms. In the test arm, *E. coli* accounted for 42/90 isolates (46.7%), followed by *Klebsiella pneumoniae* (21/90, 23.3%), *Enterococcus* spp. (13/90, 14.4%) and *Pseudomonas aeruginosa* (7/90, 7.8%). In the control arm, *E. coli* comprised 89/203 isolates (43.8%), *K. pneumoniae* (22/203, 10.8%), *Enterococcus* spp. (34/203, 16.7%) and *P. aeruginosa* (12/203, 5.9%). Less commonly isolated organisms included *Proteus*, *Morganella*, *Staphylococcus* spp., *Candida* spp. and other Gram-negative bacilli as given in **Figure 2** and **Table 2**.

**Table 2:** Culture-positive pathogen distribution.

Organism	Test arm (n=90)	Control arm (n=203)
*E. coli*	42 (46.7%)	89 (43.8%)
*K. pneumoniae*	21 (23.3%)	22 (10.8%)
*P. aeruginosa*	7 (7.8%)	12 (5.9%)
*Enterococcus* spp.	13 (14.4%)	32 (15.8%)
Other organisms	7 (7.8%)	48 (23.7%)

Overall pathogen distribution differed significantly between study arms (χ² p = 0.004). This difference appeared to reflect underlying patient case-mix rather than selective laboratory bias. In particular, the test arm demonstrated a relatively higher prevalence of *K. pneumoniae* and *P. aeruginosa*, organisms frequently associated with catheterization, prior healthcare exposure and complicated urinary infection. By contrast, the proportion of *Enterococcus* spp. were slightly more frequent in the control arm (15.8% vs 14.4%).

#### 2.2.1 MDRO burden

**Figure 3:**
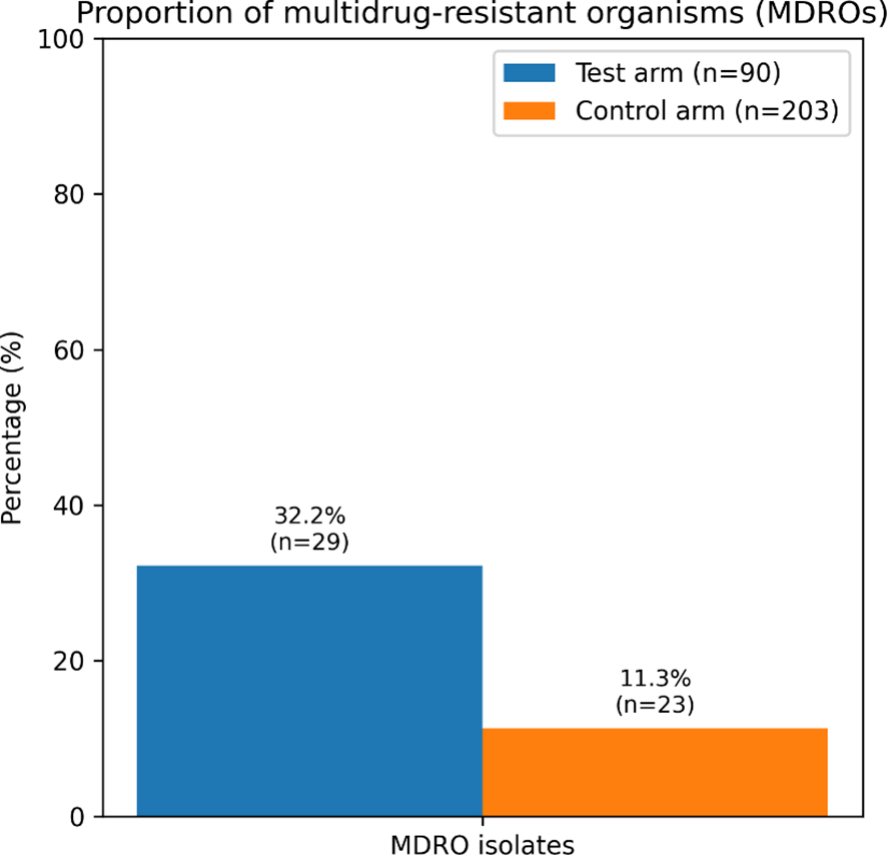
Proportion of MDRO isolates.

Across all positive isolates, 52/293 (17.7%) were multidrug-resistant. The proportion of MDROs was significantly higher in the test arm than in the control arm, with 29/90 (32.2%) versus 23/203 (11.3%), respectively (Fisher’s exact p < 0.001) as shown in **Figure 3**. The crude odds of isolating an MDRO were therefore more than three-fold higher in the test arm (OR 3.22; 95% CI 1.74–5.93). No vancomycin-resistant *Enterococcus* (VRE) was detected in either group.

This higher MDRO prevalence likely reflects the greater proportion of patients in the test arm with risk factors such as recent hospitalization, prior antimicrobial exposure and device-associated infection, rather than any adverse impact of the structured request form itself. Detailed MDRO distribution by species is presented in **Supplementary Table 9**.

### 2.3 Clinical presentation and UTI-related risk factors

Clinical presentation was fully documented in all test-arm patients due to the structured request form. ASB accounted for 84/198 (42.4%). Among the remaining 114 symptomatic cases, 71 (62.3%) presented with cystitis, 35 (30.7%) with pyelonephritis or urosepsis, and 8 (7.0%) with prostatitis or epididymo-orchitis. Catheterisation was documented in 17/198 (8.6%), recent hospitalization in 69/198 (34.8%), and prior antibiotic exposure in 50/198 (25.3%).

In the control arm, indication for urine culture could be identified in 269/286 patients (94.1%). Based on those entries, ASB accounted for 88/286 (30.8%), symptomatic cystitis for 122/286 (42.7%), and upper-tract infection for 48/286 (16.8%). However, documentation of risk factors was frequently incomplete.

ASB prevalence was significantly higher in the test arm than in the control arm (42.4% vs 30.8%; Fisher’s exact p = 0.009). Upper-tract infection was also more frequent in the test arm (17.2% vs 8.0%; Fisher’s exact p = 0.003), consistent with its clinical case-mix.

### 2.4 Management of asymptomatic bacteriuria

**Figure 4:**
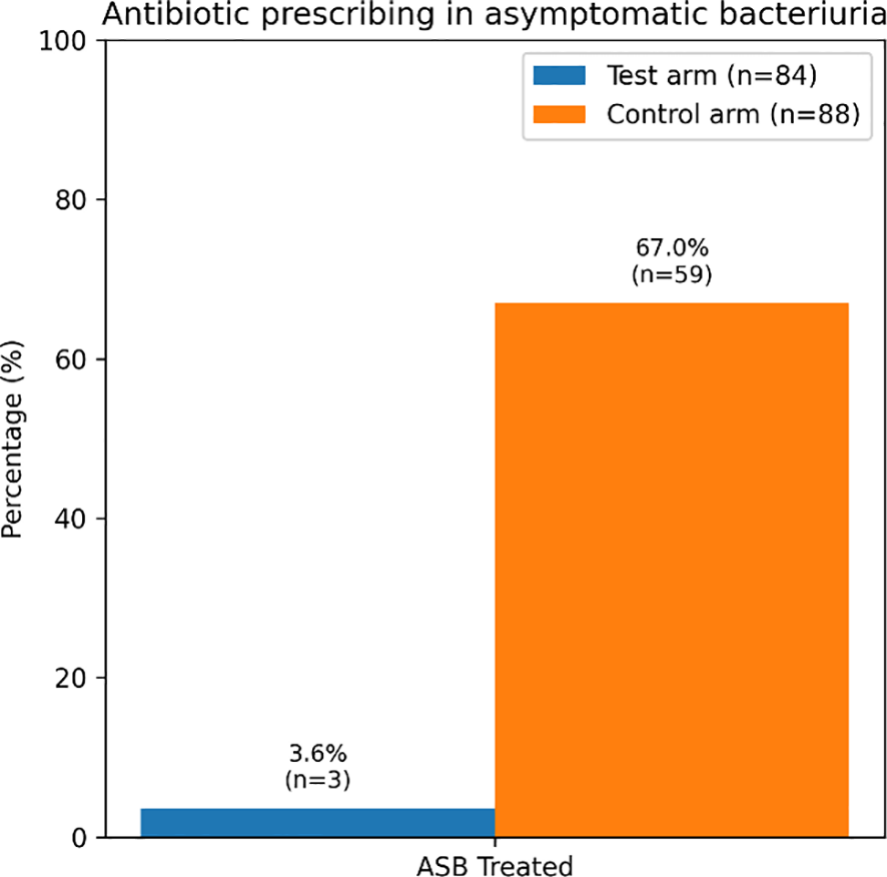
Antibiotic prescribing in asymptomatic bacteriuria.

A major difference between study arms was observed in antibiotic prescribing for ASB. In the test arm, only 3/84 ASB patients (3.6%) received antibiotics, whereas 59/88 (67.0%) were treated in the control arm (Fisher’s exact p < 0.001). None of the untreated ASB patients in the test arm developed symptomatic infection during follow-up as mentioned in **Figure 4**.

This demonstrates that structured documentation of symptom status substantially reduced overtreatment (Flores-Mireles et al., 2015).

### 2.5 Guideline-compliant therapy

Guideline compliance was assessable in 465/484 patients (missing documentation in 19 control-arm cases). Compliance was significantly higher in the test arm than in the control arm, with 146/198 (73.7%) versus 70/267 (26.2%), respectively (Fisher’s exact p < 0.001). The odds of receiving guideline-concordant therapy were almost eight-fold higher in the test arm (OR 7.90; 95% CI 5.22–11.81) as given in **Table 3**.

**Table 3:** Guideline-compliant therapy.

Outcome	Test arm (n=198)	Control arm (n=267)	p-value
Guideline compliant	146 (73.7%)	70 (26.2%)	<0.001¹

¹Fisher’s exact test.

Non-compliance in the control arm most commonly reflected unnecessary treatment of ASB or prescription of unnecessarily broad-spectrum agents. In contrast, deviations in the test arm were mainly attributable to patient-related constraints, such as delayed follow-up or inability to complete treatment.

### 2.6 Antibiotic exposure and duration of therapy

Antibiotics were prescribed in 65/198 (32.8%) test-arm patients and 59/286 (20.6%) control-arm patients (Fisher’s exact p = 0.003). Among treated patients, duration of therapy was significantly longer in the test arm (median 7 days, IQR 7–7) compared with the control arm (median 5 days, IQR 5–5) (Mann–Whitney p < 0.001). This reflected the greater proportion of complicated and upper-tract infections managed in urology and nephrology services.

### 2.7 Recurrence

**Figure 5:**
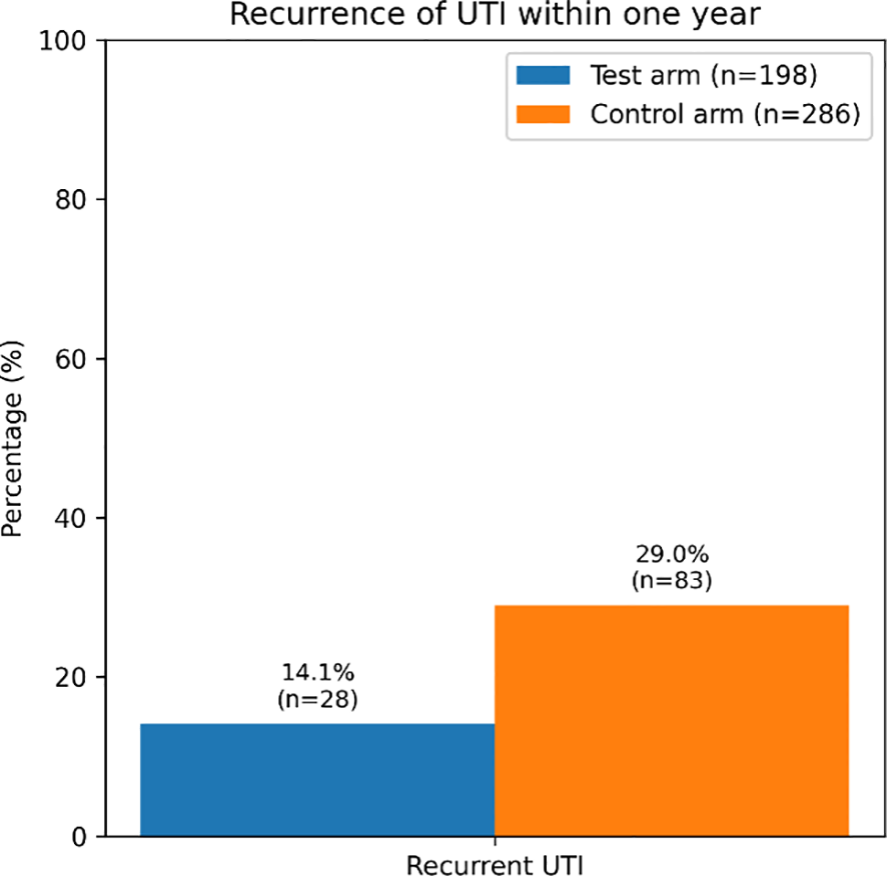
Recurrence of UTI within one year.

As shown in **Figure 5**, during one-year follow-up, recurrent UTI occurred in 28/198 (14.1%) test-arm patients and 83/286 (29.0%) control-arm patients (χ² p < 0.001). The odds of recurrence were therefore approximately halved in the test arm (OR 0.40; 95% CI 0.25–0.65). No recurrence-related complications were identified.

### 2.8 Multivariable regression

Multivariable logistic regression confirmed that, after adjusting for age, sex and clinical risk factors, use of the structured urine-culture request form was independently associated with lower odds of culture positivity, markedly higher odds of guideline-compliant prescribing and lower recurrence risk, while MDRO isolation remained primarily associated with prior healthcare exposure rather than the intervention itself.

Full model outputs are presented in **Supplementary Table 2**-**S8**.

## 3 Discussion

This single-center study evaluated the impact of a structured urine-culture request form on diagnostic clarity and antibiotic prescribing in patients investigated for urinary tract infection. Unlike many antimicrobial stewardship interventions, the form did not restrict prescribing or alter laboratory processing pathways. Instead, it enhanced the quality of routinely supplied clinical information. Despite this apparently simple modification, we observed substantial improvements in documentation completeness and antibiotic decision-making, particularly in the management of asymptomatic bacteriuria.

Incomplete or ambiguous clinical information is a recognized barrier to safe antimicrobial practice (Tamma et al., 2014; Flokas et al., 2017). In our control arm, routine request forms rarely documented key variables such as symptom status, catheterization, recent hospitalization or planned urological procedures. These omissions hinder interpretation of bacteriuria and increase the likelihood that colonization will be misclassified as infection (Flokas et al., 2017; Lee et al., 2021). By contrast, the structured request form required systematic documentation of indication and clinical risk factors, supporting more appropriate laboratory interpretation and therapeutic decision-making.

An important observation in this study was the disproportionately high rate of inappropriate treatment of asymptomatic bacteriuria in the general medicine cohort. This highlights a key opportunity for extending structured diagnostic stewardship interventions beyond specialty departments. Incorporation of guided urine culture request forms within general medicine and other high-volume departments could standardize clinical documentation, reduce diagnostic ambiguity, and improve antimicrobial decision-making. Given that general medicine often manages a heterogeneous patient population with varying levels of training and clinical exposure, such structured tools may be particularly impactful in reducing inappropriate antibiotic use at scale. Future studies should explore implementation across multiple departments to assess broader institutional benefits.

In the context of diagnostic workflow optimization, urine dipstick testing is routinely performed in our institution within the clinical pathology pathway using automated chemical strip readers; however, it was not incorporated into the present study. This study focused on culture-based microbiological evaluation and the impact of structured clinical documentation on antimicrobial decision-making. While dipstick screening is widely used in many settings, its diagnostic performance is variable and it was not included within the current diagnostic stewardship intervention. Its exclusion did not affect the primary objective of improving clinical documentation and antimicrobial prescribing. Future studies may explore integration of dipstick-based triaging with structured request forms to further optimize diagnostic pathways.

A major finding of this study was the marked reduction in unnecessary treatment of asymptomatic bacteriuria in the test arm. Multiple studies across both high-income and low- and middle-income settings report that 40–70% of patients with ASB receive antibiotics despite the absence of urinary symptoms (Leis et al., 2014; Drekonja et al., 2015; Petty et al., 2019). In a large US multicentre study including 46 hospitals, Petty et al. reported antibiotic treatment in over 80% of ASB cases, the majority without indication (Petty et al., 2019). Similar findings were observed in Veterans Affairs cohorts (Drekonja et al., 2015) and Canadian stewardship evaluations (Leis et al., 2014). These data confirm that ASB overtreatment remains a pervasive global challenge contributing significantly to avoidable antimicrobial exposure.

Our findings are consistent with this evidence base. In the control arm, where symptom documentation was inconsistent, most ASB patients were treated. In contrast, explicit documentation of symptom absence in the test arm appeared to reinforce appropriate non-treatment decisions. Importantly, withholding antibiotics was not associated with adverse outcomes, supporting long-standing guideline recommendations (Nicolle et al., 2019; Petty et al., 2019).

The structured request form also strengthened recognition of patients at increased risk of MDRO infection. Prior hospitalization, catheterization and antimicrobial exposure are established risk factors for resistant urinary pathogens (Flores-Mireles et al., 2015; Lee et al., 2021; Vaughn et al., 2021). These characteristics were relatively frequent in the urology/nephrology population and likely explain the higher crude MDRO prevalence observed in the test arm. This aligns with international evidence demonstrating that MDRO incidence reflects underlying case-mix rather than stewardship intervention itself (Tamma et al., 2019; Lee et al., 2021). Systematic capture of these risk variables enabled targeted susceptibility reporting and avoidance of unnecessarily broad therapy, reflecting a key stewardship principle. In this context, the use of department-based allocation (urology/nephrology vs general medicine) may be perceived as an unequal comparator due to differences in patient complexity and clinician expertise. However, this pragmatic design reflects real-world clinical workflows and was intended to evaluate the effectiveness of the intervention in a high-risk population, where diagnostic ambiguity and inappropriate antibiotic use are most consequential. Importantly, adjusted analyses accounting for baseline differences demonstrated consistent benefits of the intervention.

Our findings complement those of Lee et al., who demonstrated that diagnostic stewardship applied to urine microbiology workflows significantly reduced antibiotic prescribing for ASB without increasing unintended clinical events (Lee et al., 2021). Broader literature also demonstrates that structured diagnostic processes, clinical decision-support tools and improved test interpretation can reduce inappropriate prescribing and laboratory workload (Barlam et al., 2016; Cohen et al., 2016; Logan and Weinstein, 2017; Morgan et al., 2017). The present study extends this evidence by showing that a simple pre-analytical documentation tool, without workflow restriction or audit enforcement, can meaningfully influence prescribing behavior.

Importantly, inappropriate antibiotic use for suspected UTI is not limited to low-resource settings. High ASB prescribing rates and overly broad empirical therapy have been extensively documented in Europe and North America (Hooton, 2012; van Buul et al., 2012; Petty et al., 2019; Vaughn et al., 2021). For example, Vaughn et al. found that up to a quarter of all inpatient UTI-associated antibiotic exposure occurred in patients without diagnostic criteria for infection (Vaughn et al., 2021). These findings emphasize that diagnostic uncertainty is a universal driver of antimicrobial misuse. The improvements observed in this study therefore have global relevance.

In this context, an important observation in our study was the disproportionately high rate of inappropriate treatment of asymptomatic bacteriuria in the general medicine cohort. This highlights a key opportunity for extending structured diagnostic stewardship interventions beyond specialty departments. Incorporation of guided urine culture request forms within general medicine and other high-volume departments could standardize clinical documentation, reduce diagnostic ambiguity, and improve antimicrobial decision-making. Given that general medicine often manages a heterogeneous patient population with varying levels of training and clinical exposure, such structured tools may be particularly impactful in reducing inappropriate antibiotic use at scale. Future studies should explore implementation across multiple departments to assess broader institutional benefits.

Despite improved documentation, full guideline compliance was not achieved in the test arm. Clinical review showed that many deviations reflected patient-related constraints rather than prescriber knowledge gaps. This included reluctance to undergo admission-based intravenous therapy, delayed return for follow-up, early discharge before culture review, renal dysfunction limiting preferred agents, and clinical response following catheter replacement. These scenarios highlight the real-world social and physiological factors that shape stewardship outcomes (Tamma et al., 2014; Tamma et al., 2019). They also emphasize the importance of patient engagement and shared decision-making.

Clinically, this study reinforces the principle that accurate diagnosis precedes appropriate therapy. When symptom status, comorbidities and risk factors are clearly recorded, microbiologists can provide more clinically aligned reporting and prescribers can match antibiotic selection and duration to the true indication (Tamma et al., 2014; Flokas et al., 2017; Lee et al., 2021). Conversely, missing contextual information tends to bias decisions toward overtreatment. Our findings demonstrate that improved documentation alone can shift prescribing practice in a favorable direction, underlining diagnostic stewardship as a core component of antimicrobial resistance prevention strategies (Flokas et al., 2017; Logan and Weinstein, 2017; Morgan et al., 2017; Tamma et al., 2017).

### 3.1 Limitations

This was a single-center study conducted in a tertiary-care hospital with a high proportion of complex urological patients, which may limit generalizability. The study design was pragmatic and non-randomized, with allocation based on clinical departments. As a result, the test arm (urology/nephrology) included patients with a higher likelihood of complicated infections and prior healthcare exposure, whereas the control arm (general medicine) comprised relatively fewer complicated cases. Although multivariable adjustment was performed, residual confounding and selection bias cannot be fully excluded.

Documentation quality inherently differed between study arms, which may have influenced the observed prevalence of clinical risk factors. Compliance analysis in the control arm was also affected by missing data. In addition, microbiological ecology specific to the institution may limit the generalizability of resistance patterns observed in this study. Finally, formal quantification of structured request form completion (adherence) was not performed; however, training sessions and periodic reinforcement ensured high uptake in the test arm, as reflected by completeness of clinical documentation. Future studies should incorporate adherence metrics to better evaluate implementation fidelity.

## 4 Conclusion

Redesigning a urine-culture request form—a simple, low-cost, and scalable intervention—significantly improved the clarity of diagnostic information and reduced unnecessary antibiotic prescribing, particularly for asymptomatic bacteriuria, without compromising patient outcomes. These findings highlight the value of diagnostic stewardship and support the wider implementation of structured clinical documentation as part of comprehensive antimicrobial stewardship programs. This intervention is particularly relevant in low- and middle-income healthcare settings, where resource-efficient strategies to optimize antimicrobial use are critically needed.

## Data Availability

The original contributions presented in the study are included in the article/**Supplementary Material**. Further inquiries can be directed to the corresponding authors.
