# Supplementary material for: Impact of a structured urine culture request form on antimicrobial stewardship in urinary tract infections at a tertiary care hospital in India
Source: Front Antibiot. 2026 Jun 1;5:1793784. doi: 10.3389/frabi.2026.1793784 (PMC13266402; doi:10.3389/frabi.2026.1793784)
Supplement: Supplementary file 1 [file Table1.docx]

**Supplementary Figure S1. Newly designed detailed request form**


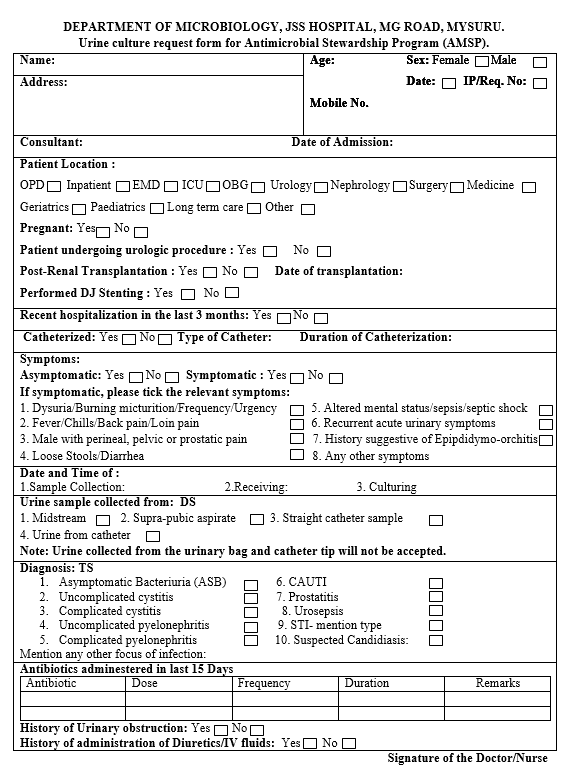


**Supplementary Figure S2. Follow-up form**


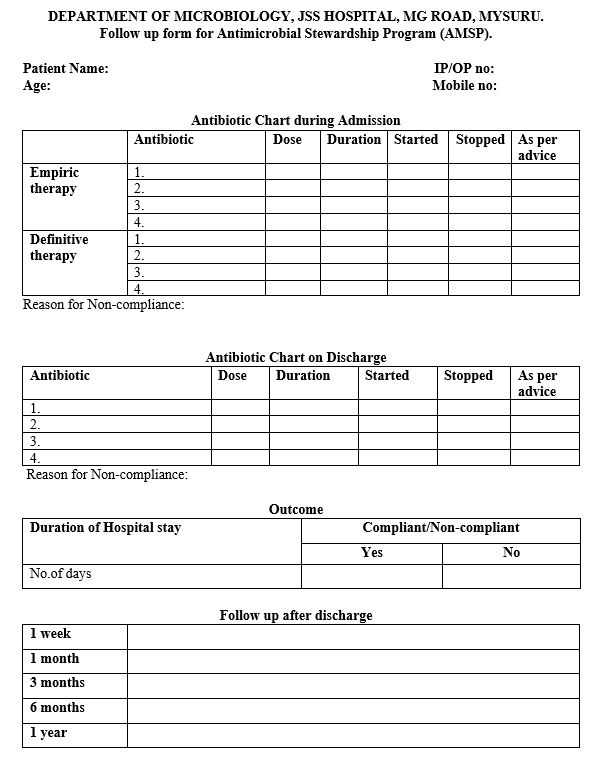


**Supplementary Table S1. Detailed summary of the clinical and microbiological**

**parameters included in the newly designed detailed request form**

| **Component** | **Details Included** |
| --- | --- |
| **MDRO Suspicion (DS)** | - Recent hospitalization  - Multiple antibiotic exposure  - Post-renal transplant  - Catheter duration  - Hemodynamic instability  → Enables susceptibility testing for higher antibiotics during initial workup. |
| **ASB Management (DS & TS)** | - Pregnancy  - Urologic procedures (pre- or post-)  - Transplant within 3 months (treat ASB)  - DJ stent in situ (ASB need not be treated) |
| **Low Count Significance (DS)** | - Obstruction and diuretic use: process low counts.  - History of gram-negative coverage: disregard Enterococcus in low counts.  - Report susceptibility only for pure cultures >10⁵ CFU/ml. |
| **Antibiotic Reporting Based on UTI Type (TS)** | - **Lower UTI/uncomplicated:** Report nitrofurantoin, fosfomycin, amikacin, cotrimoxazole, ertapenem.  - **Upper UTI/pyelonephritis or in males with perineal/pelvic/prostatic pain:** Report piperacillin-tazobactam, trimethoprim-sulfamethoxazole, imipenem, meropenem, ertapenem.  - **Sepsis/complicated UTI/recurrent UTI:** Perform sensitivity for newer BL+BLI (including ceftazidime-avibactam with aztreonam synergy). Block lower-tier antibiotics where not indicated. |
| **Sample Source & Significant Bacteriuria Thresholds (DS)** | - **Midstream urine:** ≥10⁵ CFU/ml  - **Catheter sample:** ≥10⁵ CFU/ml  - **Suprapubic aspirate:** Any growth significant (even single colony)  - **Straight catheter:** ≥10³ CFU/ml |

**Supplementary Table S2. Logistic regression analysis for predictors of urine culture positivity**

| **Variable** | **Adjusted Odds Ratio (AOR)** | **95% CI** | **p-value** |
| --- | --- | --- | --- |
| Study arm (test vs control) | 0.39 | 0.26–0.58 | <0.001 |
| Age (per year increase) | 1.01 | 1.00–1.02 | 0.11 |
| Female sex | 2.35 | 1.62–3.39 | <0.001 |
| Catheterisation | 1.89 | 0.86–4.11 | 0.11 |
| Recent hospitalisation | 1.46 | 1.00–2.13 | 0.05 |
| Complicated UTI status | 1.67 | 1.13–2.47 | 0.01 |

Hosmer–Lemeshow p = 0.42
AUC = 0.76

**Supplementary Table S3. Logistic regression analysis for predictors of MDRO isolation**

| **Variable** | **Adjusted Odds Ratio (AOR)** | **95% CI** | **p-value** |
| --- | --- | --- | --- |
| Study arm (test vs control) | 3.91 | 2.03–7.54 | <0.001 |
| Age (per year increase) | 1.01 | 0.99–1.02 | 0.28 |
| Female sex | 0.86 | 0.47–1.56 | 0.62 |
| Catheterisation | 2.43 | 0.93–6.36 | 0.07 |
| Recent hospitalisation | 1.99 | 1.06–3.73 | 0.03 |
| Complicated UTI status | 2.11 | 1.14–3.89 | 0.02 |

Hosmer–Lemeshow p = 0.57
AUC = 0.79

**Supplementary Table S4. Logistic regression analysis for predictors of guideline-compliant therapy (all evaluable patients)**

| **Variable** | **Adjusted Odds Ratio (AOR)** | **95% CI** | **p-value** |
| --- | --- | --- | --- |
| Study arm (test vs control) | 7.90 | 5.22–11.81 | <0.001 |
| Age (per year increase) | 1.00 | 0.99–1.01 | 0.88 |
| Female sex | 1.21 | 0.86–1.72 | 0.28 |
| Catheterisation | 0.91 | 0.48–1.72 | 0.78 |
| Recent hospitalisation | 0.94 | 0.66–1.33 | 0.72 |
| Complicated UTI status | 0.89 | 0.63–1.25 | 0.50 |

Hosmer–Lemeshow p = 0.63
AUC = 0.82

**Supplementary Table S5. Logistic regression analysis for predictors of UTI recurrence within one year**

| **Variable** | **Adjusted Odds Ratio (AOR)** | **95% CI** | **p-value** |
| --- | --- | --- | --- |
| Study arm (test vs control) | 0.46 | 0.28–0.73 | 0.001 |
| Age (per year increase) | 1.01 | 1.00–1.02 | 0.07 |
| Female sex | 1.38 | 0.91–2.07 | 0.13 |
| Catheterisation | 1.84 | 0.88–3.83 | 0.10 |
| Recent hospitalisation | 1.67 | 1.10–2.54 | 0.02 |
| Complicated UTI status | 1.92 | 1.22–3.03 | 0.005 |

Hosmer–Lemeshow p = 0.71
AUC = 0.74

**Supplementary Table S6. Linear regression model for predictors of days-of-therapy (DOT) among patients receiving antibiotics**

| **Variable** | **β-coefficient (days)** | **95% CI** | **p-value** |
| --- | --- | --- | --- |
| Study arm (test vs control) | +2.14 | +1.28 to +2.99 | <0.001 |
| Age (per year increase) | +0.02 | –0.01 to +0.04 | 0.19 |
| Female sex | –0.34 | –1.12 to +0.44 | 0.39 |
| Catheterisation | +0.88 | –0.54 to +2.31 | 0.22 |
| Recent hospitalisation | +0.95 | +0.21 to +1.69 | 0.01 |
| Complicated UTI status | +1.73 | +0.98 to +2.49 | <0.001 |

Model R² = 0.41

**Supplementary Table S7. Logistic regression analysis for predictors of receiving antibiotics (any indication)**

| **Variable** | **Adjusted Odds Ratio (AOR)** | **95% CI** | **p-value** |
| --- | --- | --- | --- |
| Study arm (test vs control) | 1.88 | 1.24–2.81 | 0.003 |
| Age (per year increase) | 1.01 | 1.00–1.02 | 0.09 |
| Female sex | 1.34 | 0.92–1.96 | 0.12 |
| Catheterisation | 1.96 | 0.86–4.49 | 0.11 |
| Recent hospitalisation | 1.59 | 0.94–2.70 | 0.09 |
| Complicated UTI status | 1.88 | 1.10–3.21 | 0.02 |

Hosmer–Lemeshow p = 0.47
AUC = 0.73

**Supplementary Table S8A. Sensitivity analysis: predictors of guideline-compliant therapy restricted to culture-positive symptomatic UTI**

| **Variable** | **Adjusted Odds Ratio (AOR)** | **95% CI** | **p-value** |
| --- | --- | --- | --- |
| Study arm (test vs control) | 5.91 | 3.49–9.99 | <0.001 |
| Age (per year increase) | 0.99 | 0.98–1.01 | 0.24 |
| Female sex | 1.18 | 0.71–1.96 | 0.52 |
| Complicated UTI | 0.89 | 0.51–1.55 | 0.67 |
| Catheterisation | 0.74 | 0.29–1.87 | 0.52 |
| Recent hospitalisation | 0.95 | 0.55–1.63 | 0.84 |

Hosmer–Lemeshow p = 0.69
AUC = 0.83

**Supplementary Table S8B. Sensitivity analysis: predictors of recurrence restricted to culture-positive symptomatic UTI**

| **Variable** | **Adjusted Odds Ratio (AOR)** | **95% CI** | **p-value** |
| --- | --- | --- | --- |
| Study arm (test vs control) | 0.51 | 0.30–0.89 | 0.02 |
| Age (per year increase) | 1.01 | 0.99–1.02 | 0.09 |
| Female sex | 1.34 | 0.77–2.32 | 0.29 |
| Complicated UTI | 1.88 | 1.10–3.21 | 0.02 |
| Catheterisation | 1.96 | 0.86–4.49 | 0.11 |
| Recent hospitalisation | 1.59 | 0.94–2.70 | 0.09 |

Hosmer–Lemeshow p = 0.47
AUC = 0.73

**Supplementary Table S9. Detailed pathogen distribution and resistance profiles in test and control arms**

| **Pathogen** | **Resistance Category** | **Test Arm (n=90)** | **Control Arm (n=203)** |
| --- | --- | --- | --- |
| *E. coli* | MDR | 11 (12.2%) | 12 (5.9%) |
|  | Non-MDR | 29 (32.2%) | 67 (33.0%) |
|  | Sensitive to all | 2 (2.2%) | 10 (4.9%) |
| *K. pneumoniae* | MDR | 13 (14.4%) | 9 (4.4%) |
|  | Non-MDR | 7 (7.8%) | 10 (4.9%) |
|  | Sensitive to all | 1 (1.1%) | 3 (1.5%) |
| *P. aeruginosa* | MDR | 5 (5.6%) | 2 (1.0%) |
|  | Non-MDR | 1 (1.1%) | 9 (4.4%) |
|  | Sensitive to all | 1 (1.1%) | 1 (0.5%) |
| *Enterococci* | MDR | 0 | 0 |
|  | Non-MDR | 11 (12.2%) | 21 (10.3%) |
|  | Sensitive to all | 2 (2.2%) | 11 (5.4%) |
| Other pathogens¹ | Total | 7 (7.8%) | 48 (23.7%) |

¹ Other pathogens include:
*Acinetobacter baumannii, Burkholderia cepacia, Candida tropicalis, Candida albicans, Candida glabrata, Candida kefyr, Candida ciferrii, Staphylococcus haemolyticus, Staphylococcus epidermidis, Staphylococcus aureus, Streptococcus species, Citrobacter koseri, Enterobacter cloacae, Staphylococcus saprophyticus, Morganella morganii, Proteus mirabilis, Proteus putida.*
